# Supplementary figures and images for: Auxin Dynamics and Transcriptome–Metabolome Integration Determine Graft Compatibility in Litchi (Litchi chinensis Sonn.)
Source: Int J Mol Sci. 2025 Apr 29;26(9):4231. doi: 10.3390/ijms26094231 (PMC12072320; doi:10.3390/ijms26094231)

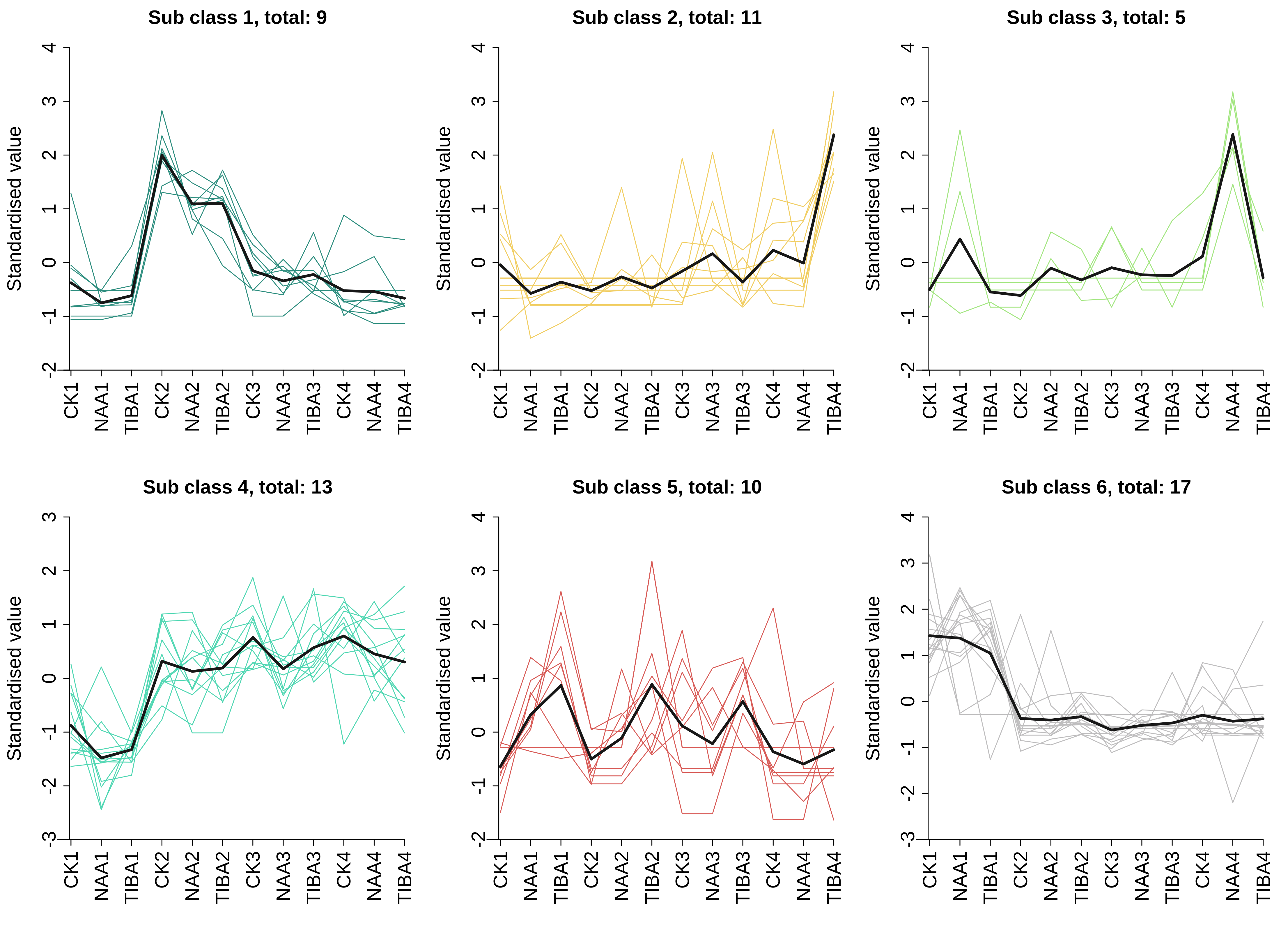

Supplement: Supplementary file 1 [file ijms-26-04231-s001.zip › Figure S1.png]

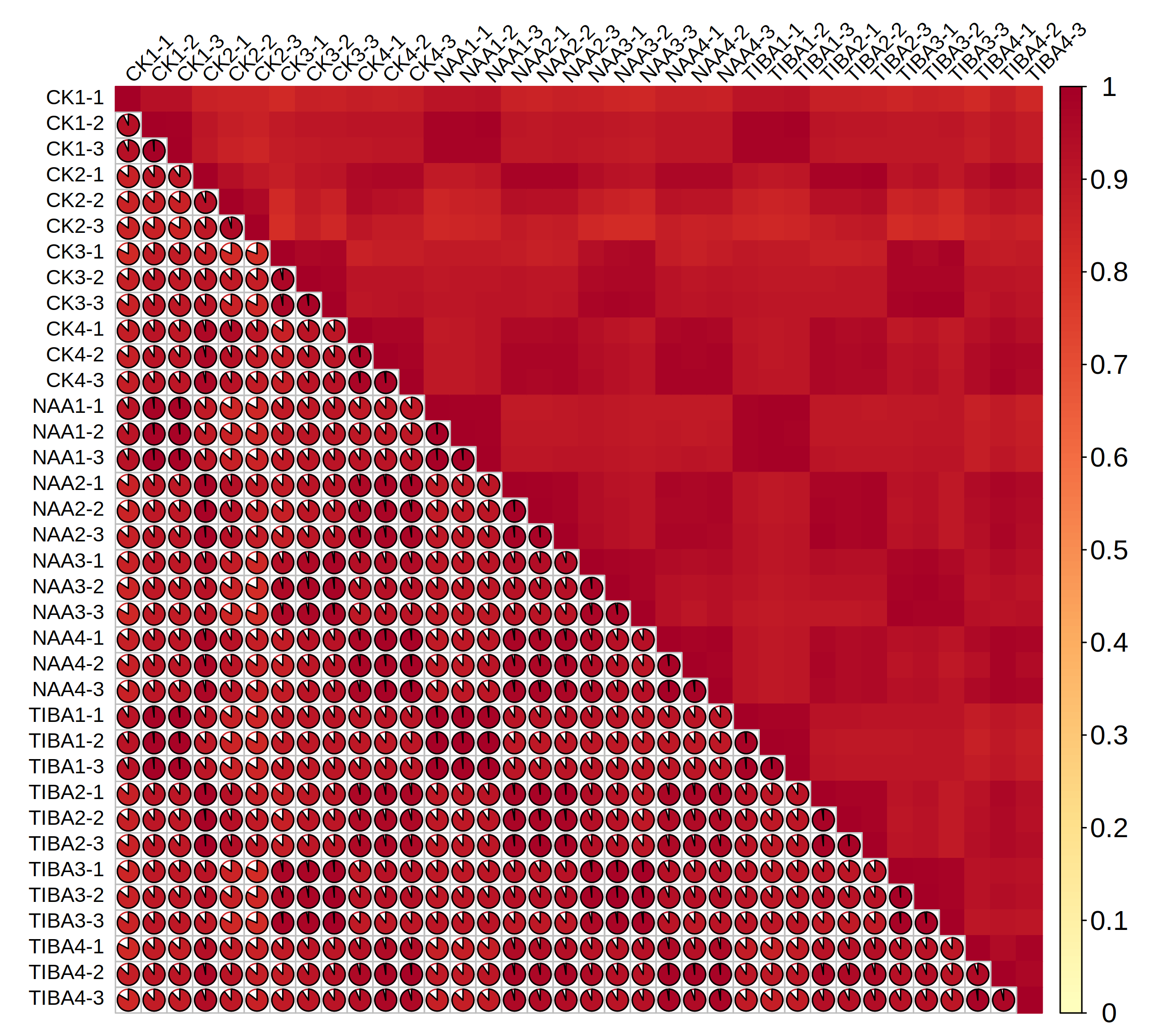

Supplement: Supplementary file 1 [file ijms-26-04231-s001.zip › Figure S2.png]

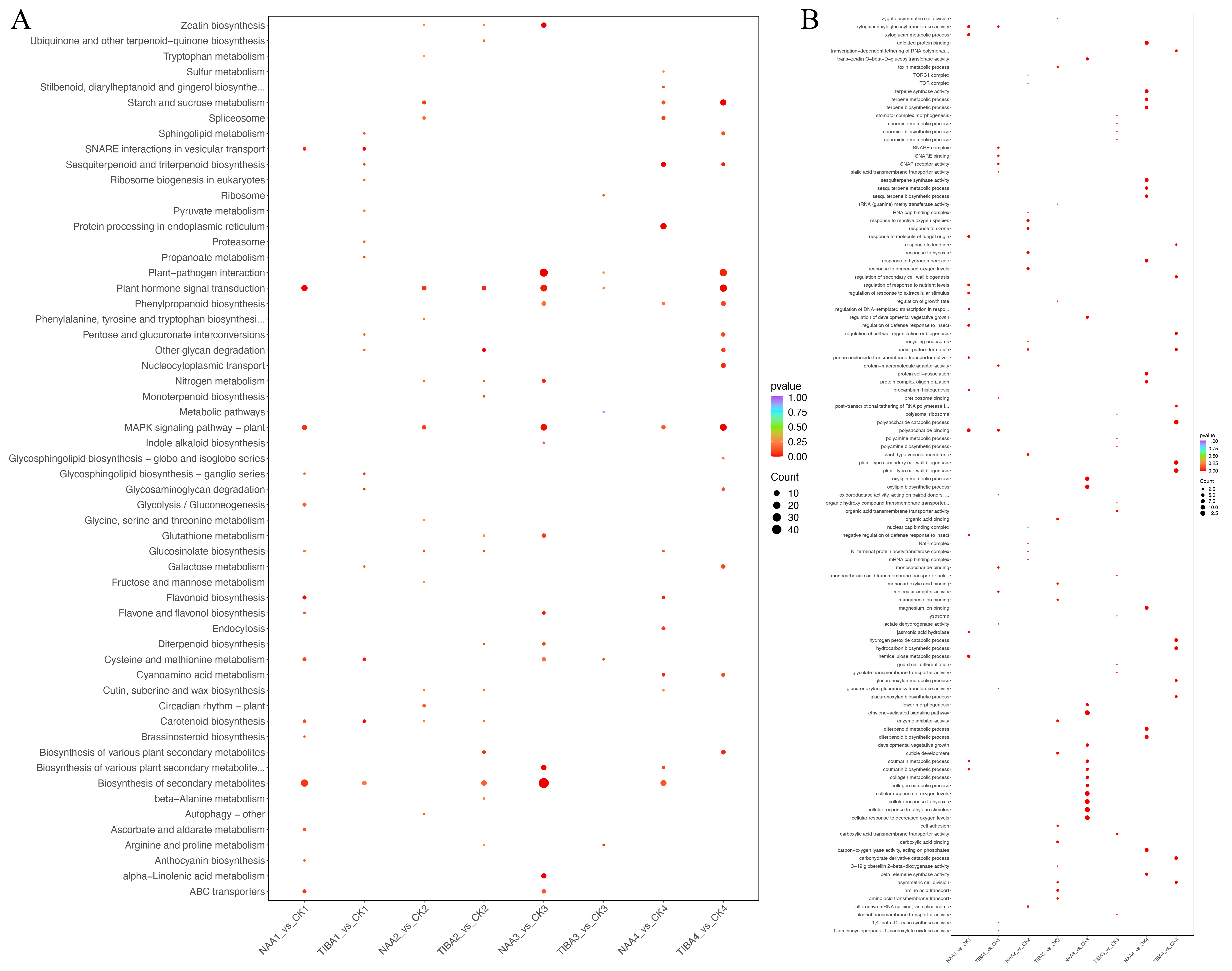

Supplement: Supplementary file 1 [file ijms-26-04231-s001.zip › Figure S3.jpg]

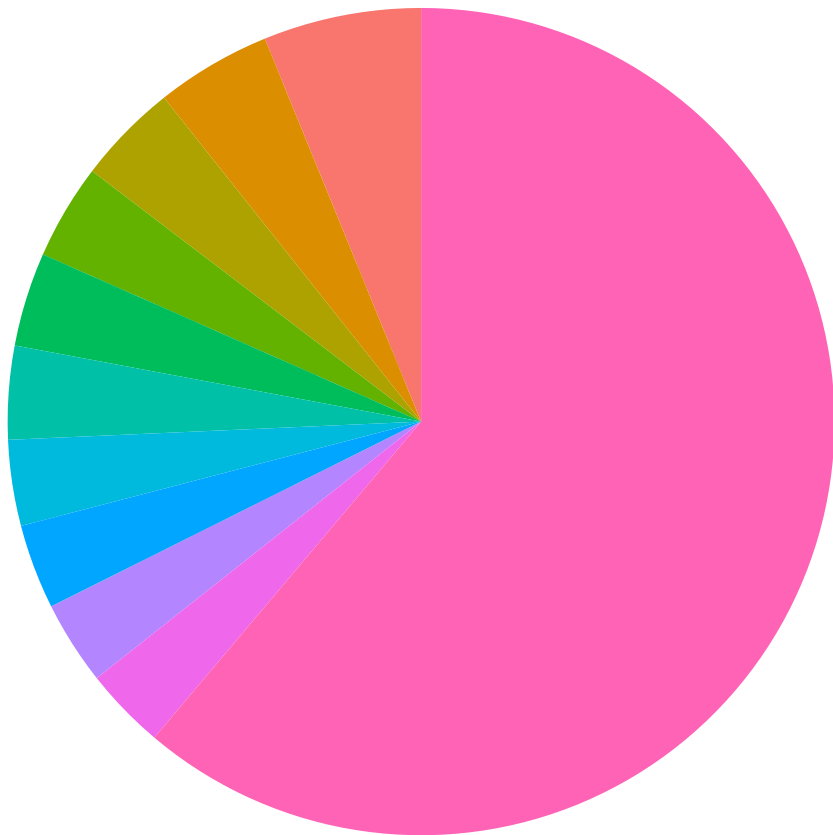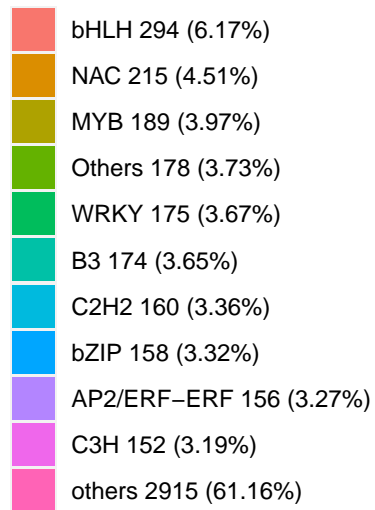

Supplement: Supplementary file 1 [file ijms-26-04231-s001.zip › Figure S4.pdf]
